# 3D analysis of soft tissue dimensional changes after dental implant placement with butt-joint vs. conical connection: a 12-month randomized control trial

**DOI:** 10.1186/s40729-024-00585-4

**Published:** 2024-12-19

**Authors:** André-Joubin Derakhshani, Florian Beuer, Mats Wernfried Heinrich Böse, Insa Herklotz, Alexey Unkovskiy

**Affiliations:** 1 Department of Prosthodontics, Geriatric Dentistry and Craniomandibular Disorders, Charité - Universitätsmedizin Berlin, Corporate Member of Freie Universität Berlin, Humboldt- Universität zu Berlin, Aßmannshauser Str. 4-6, 14197 Berlin, Germany; 2 Gröpelinger Heerstraße 406, Mund. Kiefer. Gesicht. Bremen, 28239 Bremen, Germany; 3 Zahnarztpraxis Amalienpark – Dr. Herklotz & Dr. Thiele, Amalienpark 1, 13187 Berlin, Germany; 4 Department of Dental Surgery, Sechenov First Moscow State Medical University, Bolshaya Pirogovskaya Street, 19с1, Moscow, 119146 Russia

**Keywords:** Dental implants, Emergence profile, Connective tissue, Periimplant soft tissue, Butt-joint, Conical implant-abutment connection

## Abstract

**Purpose:**

to quantify the soft tissue dimensional changes after single-gap implant placement, during healing abutment and crown delivery phase for butt-joint and conical implant-abutment connection type.

**Methods:**

forty patients were enrolled in the study and received randomly allocated implants with butt-joint and conical implant-abutment connection type. A standard healing abutment was placed after 6 months for two weeks. The definitive screw retained full-ceramic crowns were manufactured in a digital workflow. The soft tissue profile was digitized using IOS on following stages: pre-op, immediately, two, 7 and 14 days post-op, pre-exposure, immediately after exposure, two weeks after exposure (pre-delivery), immediately after crown delivery, 6 and 12 months after delivery. The intraoral scans were matched in the metrology software (Geomagic Control X). The mean maximum and mean average differences in mm were gathered to assess the soft tissues change. Various anamnesis parameters have been taken into account.

**Results:**

the conical connection implant system exhibited more soft tissue gain and less recession, compared to the butt-joint connection type within the 12 months follow-up period. A higher loss of soft tissue was observed in the distal papilla than in the mesial one.

**Conclusions:**

the implant-abutment connection type may influence the reaction of peri-implant soft tissue within the 12 months follow-up period.

## Background

Dental implants are a favoured and effective way to replace missing teeth, which significantly improves patients’ quality of live [1, 2]. In single tooth gaps it commonly involves a minor surgical procedure with the advantage that tooth structure of neighbouring teeth can be preserved [3]. 

The way a dental implant interacts with the surrounding soft tissue can have a significant impact on the overall aesthetics of the treatment outcome [4–7]. One of the primary factors that contribute to the aesthetics of the implant-supported restorations is the emergence profile, which refers to the way the implant-supported restorations emerge from the implant and gumline [4]. The emergence profile should closely match that of a natural tooth to create a seamless transition between the implant and surrounding teeth [4, 5, 8]. The sufficient amount of surrounding soft tissue play a crucial role in long-term success and stability of dental implants [5, 6, 9]. 

The connection type between the implant and abutment may also influence the soft tissue profile [10, 11]. Thus, the connection with an integrated platform switch may be beneficial with regards to the soft tissue gain [11, 12]. The butt-joint connection, also known as a flat-to-flat connection, involves the direct contact of the abutment and implant surfaces without any tapering and most commonly without any platform switch. has been proven that the conical connection with integrated platform switch is beneficial with regards to the crestal bone stability due to a better load distribution on the peri-implant tissue and interface sealing. However, the exact reaction of soft tissue on the implant-abutment connection type was not quantified as for now.

With the advent of intraoral scanning it became possible to record the soft tissue profile during the follow-up and trace its dimensional changes after various surgical or prosthetic interventions by matching the gathered 3D datasets [13]. This strategy was successfully used to assess the soft tissue gain by using connective tissue grafts and free gingival grafts [14–17]. 

The aim of the present study was to quantify the soft tissue dimensional changes after implant placement, during healing abutment and crown delivery phase for two different implant types. The null hypothesis of the study was that there would be no difference between conical and butt-joint implant-abutment connection types with regards to reaction and dimensional changes of peri-implant soft tissue.

## Methods

### Patients’ recruitment and study design

**Fig. 1:**
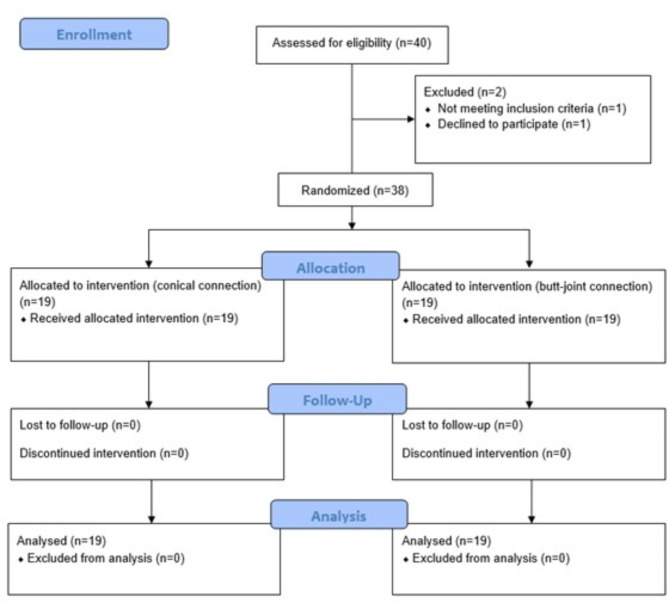
CONSORT diagram

This study was designed as parallel-arm, active control, examiner masked, randomized controlled clinical trial investigating the soft tissue dimensional changes using conical and butt-joint implant-abutment connection type. The study was performed in accordance to the guidelines presented in the Helsinki Declaration of Ethical Principles for Medical Research. The manuscript was organized following the Consolidated Standards of Reporting Trials guidelines (CONSORT) (Fig. 1). All patients provided informed written consent to participate in this study. The study protocol was approved by the ethical committee of Charité University Hospital (application number: EA4_111_19). The study was registered in the ClinicalTrial.gov with identifier NCT06627023.

The inclusion criteria were defined as follows: presence of a single tooth gap, completion of conservative and periodontal treatments, presence of diagnostic preoperative models and a recent panoramic radiograph not older than 6 months. The exclusion criteria were abnormal jaw anatomy (e.g., significant bone defects or malformations) and insufficient bone volume, abnormal bone conditions (such as cysts or tumors), abnormal findings in the oral mucosa (e.g., oral lesions or mucosal diseases), untreated periodontal disease, acute inflammatory diseases, pregnancy, temporarily contraindicated medication use, psychological contraindications such as substance abuse (alcohol or drugs), poor compliance, and general medical contraindications or conditions. All gathered datasets were anonymized and each participant was assigned a study number. A randomization was performed regarding the implant-abutment connection type, using a computer-generated list. Allocation concealment was ensured to prevent any bias through a secure online randomization system that provided assignment information only to authorized personnel at the time of patient enrolment. All enrolled patients were anonymized and each participant was assigned a study number. A priori power analysis based on the results of a previous study of Ramanauiskaite et al. revealed that 15 patients in the group is sufficient to detect difference of 0.36 mm with statistical power of 95% and α = 0.05.^17^

### Virtual implant planning

The patients who were admitted to participate in the study were guided through the following scheme: initially, cone beam computed tomography (CBCT) of respective jaws with an individually adjusted volume of mostly 100 × 50 mm (Veraviewepocs 3D R100, Morita, Kyoto, Japan) was performed and exported in Digital Imaging and Communications in Medicine (DICOM) format. Subsequently impressions with a vinyl polyether silicone (EXA’lence; GC, Tokyo, Japan) were required and a master model was cast from type IV plaster (SHERAHARD-ROCK, SHERA Werkstoff-Technologie GmbH & Co, Lemförde, Germany). Thereafter, analogue wax-ups of the missing teeth were performed. Both, initial situation and wax-ups were digitized using a laboratory scanner (D2000, 3Shape, Copenhagen, Denmark) and exported in Standard Tessellation/Triangulation Language (STL).

Virtual implant planning was performed by matching of DICOM and STL datasets from digitized master models and wax-ups in two different implant planning software. SMOP software (Swissmedia AG, Baar, Switzerland) was used for butt-joint group (CAMLOG SCREW LINE Promote plus) and coDiagnostiX^®^ (Dental Wings GmbH, Chemnitz, Germany) for conical connection group (Straumann Bone Level Tapered). Surgical guides were designed by specialist from respective companies and STL files were provided for 3D printing with stereolithography (SLA) technique (Form 3, Formlabs, Sommerville, Massachutes, USA).

### Implant placement

In the butt-joint group the CAMLOG SCREW LINE Promote plus implants were placed. In the conical group Straumann Bone Level Tapered were placed. In both cases the implants were placed with the 30–40 Ncm torque. The supracrestal dimension was respected, so that the distance between the platform and soft tissue margin was at least 4 mm. In case of butt-joint connection, if the implant was placed subcrestaly, the surrounding bone was profiled using a special bur, in order to avoid bone remodelling in the healing phase. In case of conical connection, the subcrestaly placed implants were left as is. All implants had at least 1.5 mm of surrounding bone. After dental implant placement, a closed healing approach was applied. Wounds were stitched with one horizontal mattress suture (Prolene 5.0, Johnson and Johnson, Norderstedt, Germany) and at least three single sutures (Prolene 6.0, Johnson and Johnson, Norderstedt, Germany).

### Implant exposure

Second stage surgery was performed three to six months after implant installation. Prefabricated healing abutments were placed (CAMLOG wide body healing abutment and Straumann conical healing abutment). No suturing was performed after uncovering. Two weeks after exposure the positions of dental implants were scanned using an intraoral scanner (IOS) (Trios3, 3Shape, Copenhagen, Denmark) and sent to the in-house dental laboratory of Charité University Hospital. The final screw-retained crowns were made from a high-strength glass-ceramic (Suprinity^®^, Vita Zahnfabrik Bad Säckingen, Germany) and were screwed with either a torque of 20 NCm (CAMLOG) or 35 NCm (Straumann) followed by closure with teflon tape and composite.

### Follow-up and 3D data gathering

**Fig. 2:**
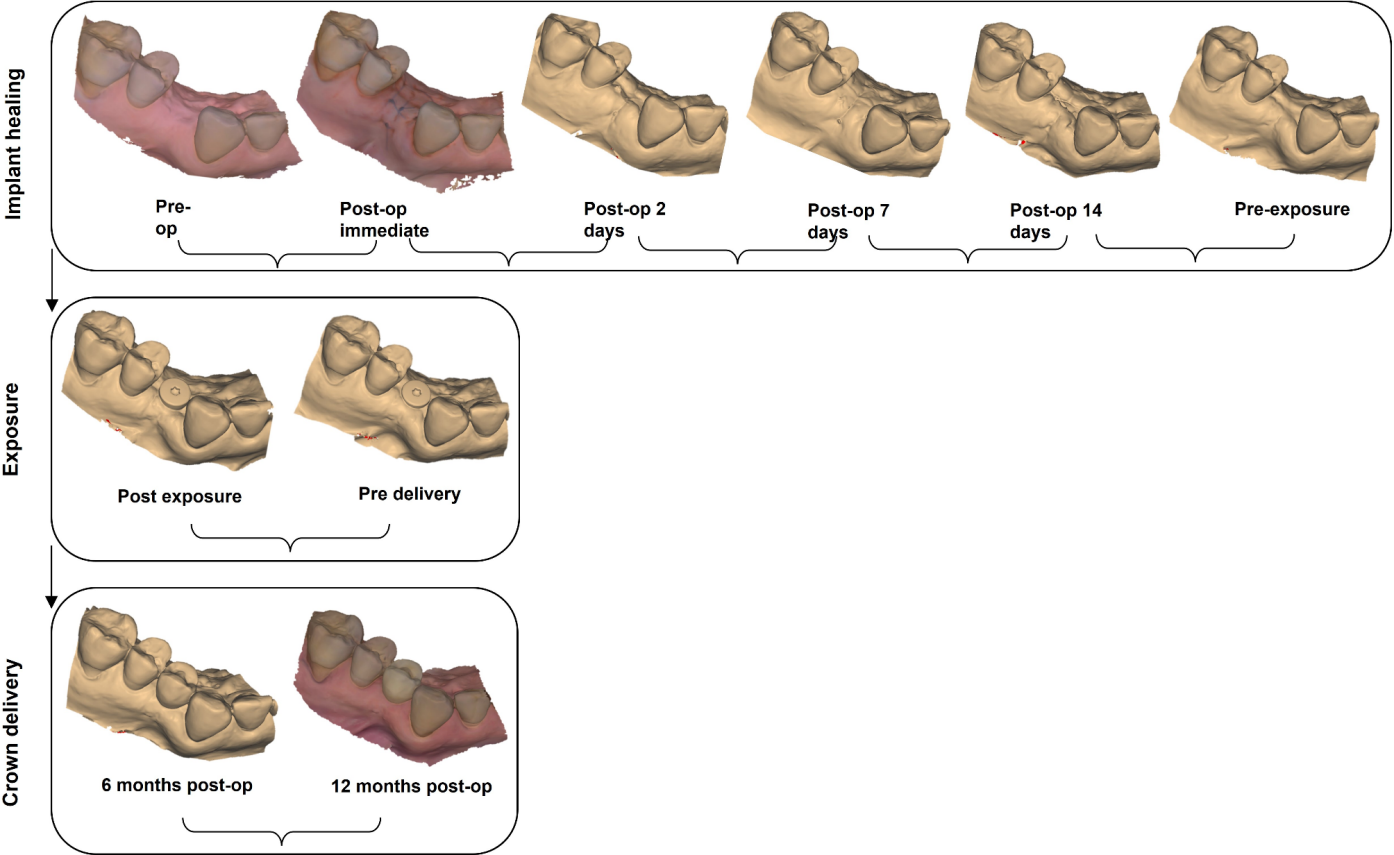
Timeline of intraoral scans divided into three main groups: implant healing phase, exposure with healing abutment placement, and crown delivery. For each group an STL data was obtaines using IOS

All intraoral scans were performed by a single calibrated operator with the same IOS (Trios3, 3Shape, Copenhagen, Denmark). The first scan was made before surgery, then immediately after implant placement and at key intervals during the healing process (e.g., 14 days post-op, immediately after exposure), two weeks after exposure (pre-delivery), immediately after delivery of the final crown and at 6 and 12 months after implant placement (Fig. 2). The scans were performed in adherence to the manufacturers recommended scanning strategy [18]. At any stage of follow-up intraoral scans were performed prior to local anaesthesia in order to exclude any influence of the injection on the soft tissue volume. The follow-up period was measured from the day of implant insertion, establishing a maximum follow-up of 12 months after implant placement. The healing abutment phase lasted an average of 2.3 months, and an average of 7 months elapsed between the integration of the final crown and the last scan.

### Gathered data post-processing

A trained and calibrated specialist performed data post-processing of the STL datasets. The STL files were trimmed to focus on the peri-implant soft tissue, excluding the healing abutment and implant crowns using Blender software (Blender Foundation, Amsterdam, Netherlands) to avoid interference in the matching process. Two STL datasets from each matching group (Fig. 2) were imported into Geomagic Control X (3D Systems, Morrisville, North Carolina) for analysis.

### Data analyses

Two of the processed STL datasets of a corresponding matching group (Fig. 2) were imported into the metrology software Geomagic Control X (3D Systems, Morrisville, North Carolina). The matching process had two stages. First, a pre-matching step was conducted using the “transform fit” tool, which aligned the datasets by selecting four distinctive points on the adjacent teeth. Then, the segmented teeth were used as a reference surface for final alignment using the “best fit” tool.

**Fig. 3:**
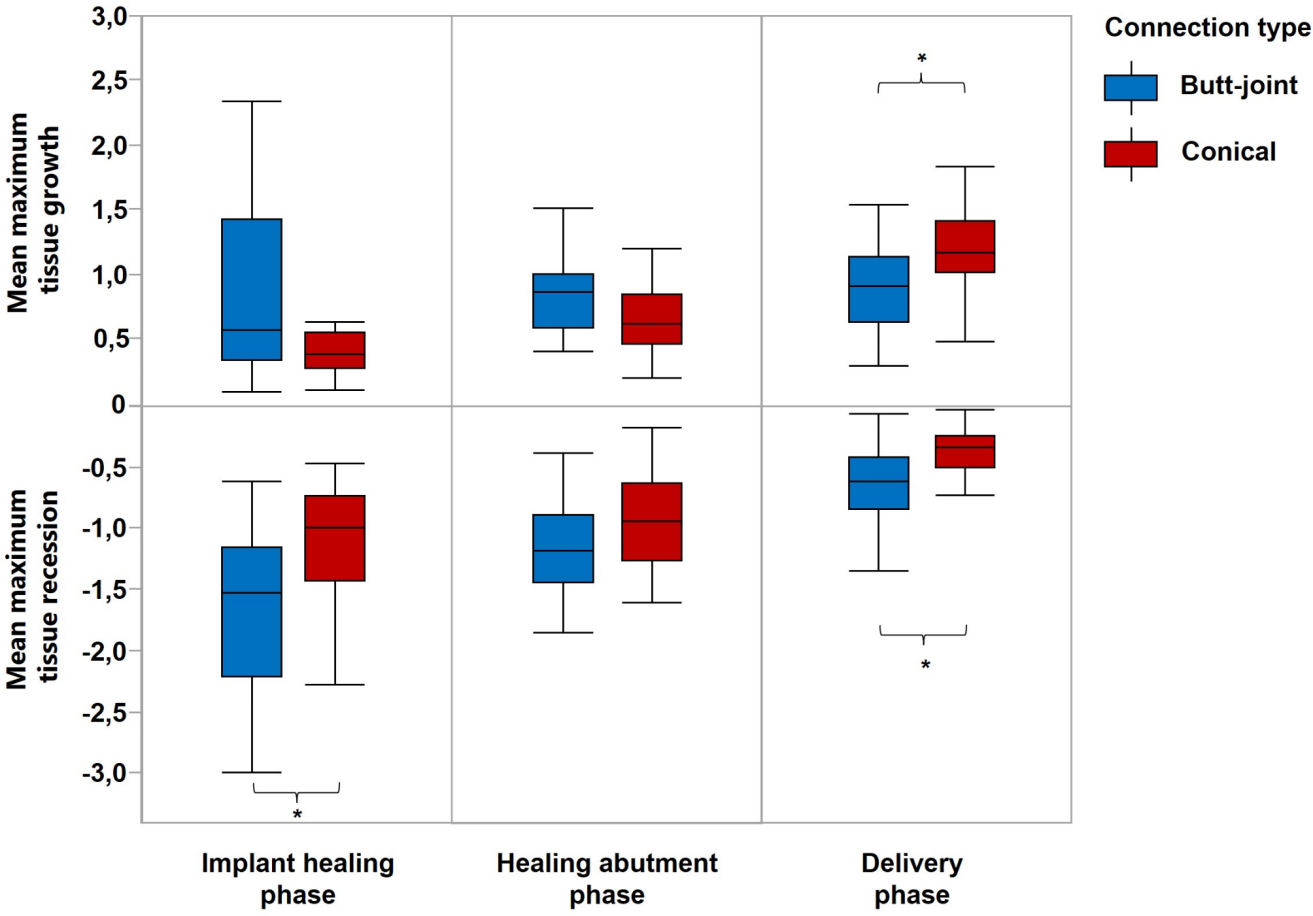
Primary outcome of tghe study: sigificantly higher soft tissue groth in conical connection type compared to butt-joint

Next, the differences between the STL datasets around the implant site were digitally measured (Fig. 3). The analyzed region extended 4 mm caudally from the middle of the adjacent teeth, with a straight-line tolerance of ± 0.1 mm, ensuring measurements remained within the attached gingiva. A heat map was generated to qualitatively show positive and negative deviations in soft tissue relief. While linear measurements were primarily used, this method was deemed appropriate for the objectives of the study. For the quantitative analysis, the maximum and average mean differences in the marked areas were measured (in mm).

For the qualitative analysis a heat map was generated to depict the negative and positive deviations regarding the soft tissue relief. For the quantitative analysis in the marked area the following parameters were obtained and used to compare the soft tissue relief: maximum mean difference and average mean difference in mm.

**Fig. 4:**
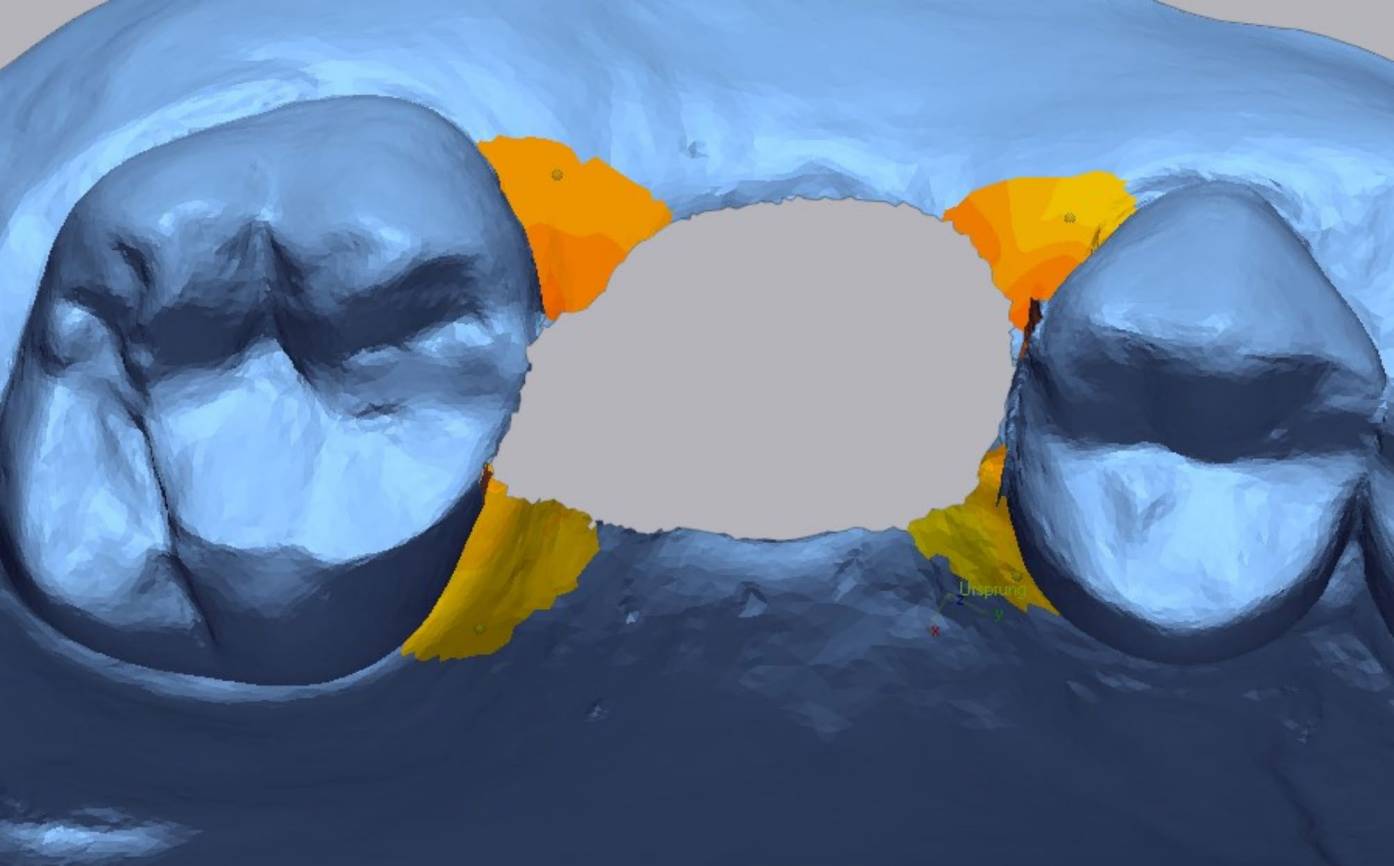
The examined area was extended from the papilla tip of the respective neighbouring tooth to 2 mm caudal and ventro-dorsal to the sulcus of the implant crown

In order to analyse the papillae, the selected region was divided into 4 areas: mesio-vestibular, disto-vestibular, disto-oral, and mesio-oral. Each papilla was defined by a line extending 2 mm caudally from the top of the respective papilla (Fig. 4). From this point, the area in the ventro-dorsal direction was selected, reaching up to the sulcus of the implant crown and the respective neighbouring tooth. Subsequently, a 3D comparison was conducted using the same method as for analysing the peri-implant soft tissue as described above.

### Special anamnesis

For all enrolled patients a detailed medical historywas collected, including following parameters: sex, region, brushing, mouth rinsing, flossing, alcohol consumption, stress, periodontitis therapy, gingival biotype.

### Statistical analyses

All gathered data were statistically analysed in JMP 14 (SAS Corp., Heidelberg, Germany). Firstly, soft tissue measurements were tested for normal distribution by goodness of fit with the Shapiro-Wilk test. For non-normally distributed data the statistical difference was analysed by using Wilcoxon test. The threshold for significance was defined as a p-value less than 0.05.

## Results

### Participant flow and recruitment

Patient recruitment phase started in November 2021 and ended in December 2022. 40 patients were screened and finally, a total of 38 patients enrolled to the clinical trial having fulfilled all inclusion criteria. 19 patients were allocated to conical group (Straumann) and 19 patients – to butt-joint group (Camlog). The groups were comparable in age and gender. All 38 included patients were treated and subsequently completed the study and follow-up.

### Primary outcomes

In the implant healing phase, the butt-joint connection demonstrated significantly higher soft tissue recession, compared to conical connection. In the healing abutment phase, no significant differences were observed between two study groups. In the delivery phase significantly higher soft tissue growth and less recession were observed in the conical connection group compared to the butt-joint group (Fig. 4).

**Fig. 5:**
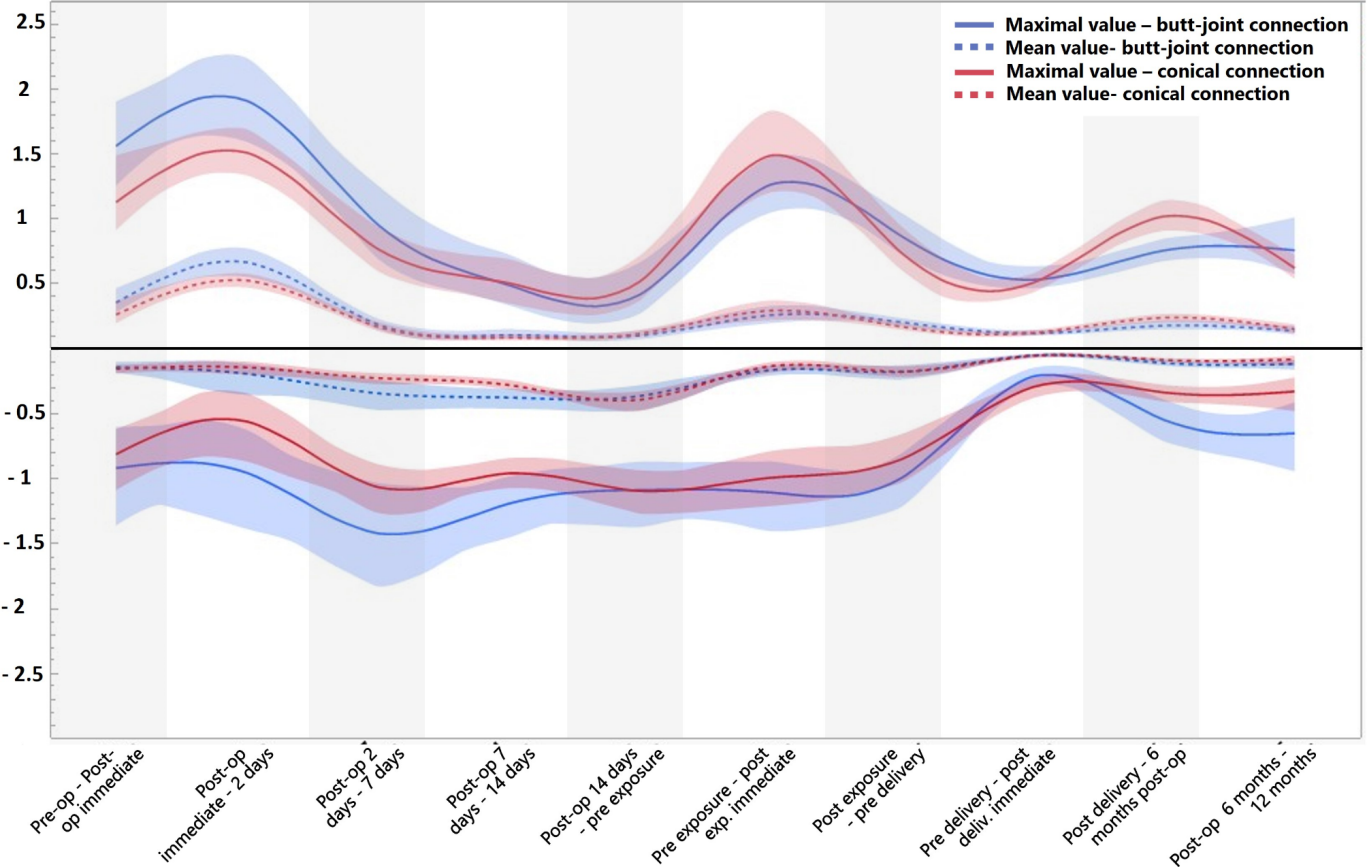
Timeline of the dimensional changes of peri-implant soft tissue. Each period reflects the mean maximum (solid line) and mean average (dashed line) difference between the two neighbour clinical steps

**Fig. 6:**
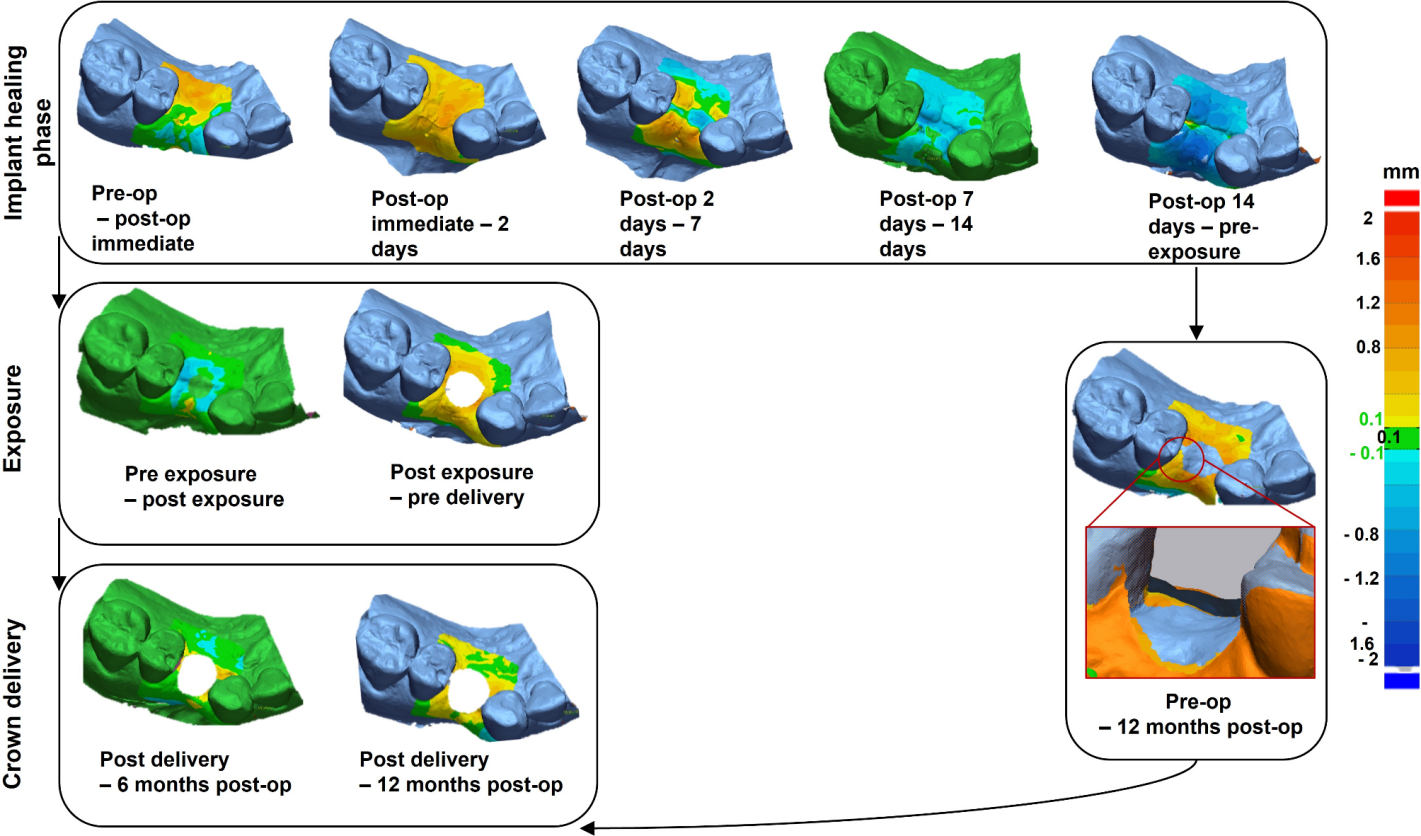
Timeline of intraoral scans divided into three main groups: implant healing phase, exposure with healing abutment placement, and crown delivery. Each group describes a match between two neighbouring STL datasets in metrology software (Geomagic Control X). The final image demonstrates the difference between the pre-op and 12 post-op situation. Note the sculpted papillae. The tolerance threshold was set on ± 0.1 mm. The soft tissue grouth is marked with yellow to red colors and recession with blue colors

The soft tissues change around dental implants are demonstrated in quantitative (Fig. 5) and qualitative manner (Fig. 6). It can be clearly seen that postoperative swelling reached a maximum on the second day after surgery, significantly decreased on the seventh day and disappeared on the fourteenth day in case of both conical and butt-joint implant connection type. Between the second and seventh day after surgery there has also been the most recession. The pike that can be seen on the stage pre-exposure – post-exposure refers to the mechanical push of the soft tissue through the healing abutment. During the healing abutment stage, the tissue demonstrated a little recession in both study groups. With regards to crown delivery the conical implant system exhibited a higher soft tissue growth and less recession compared to the butt-joint connection type. The tendency of increased tissue recession for the butt-joint group remains on the 6 to 12 months stage.

**Fig. 7:**
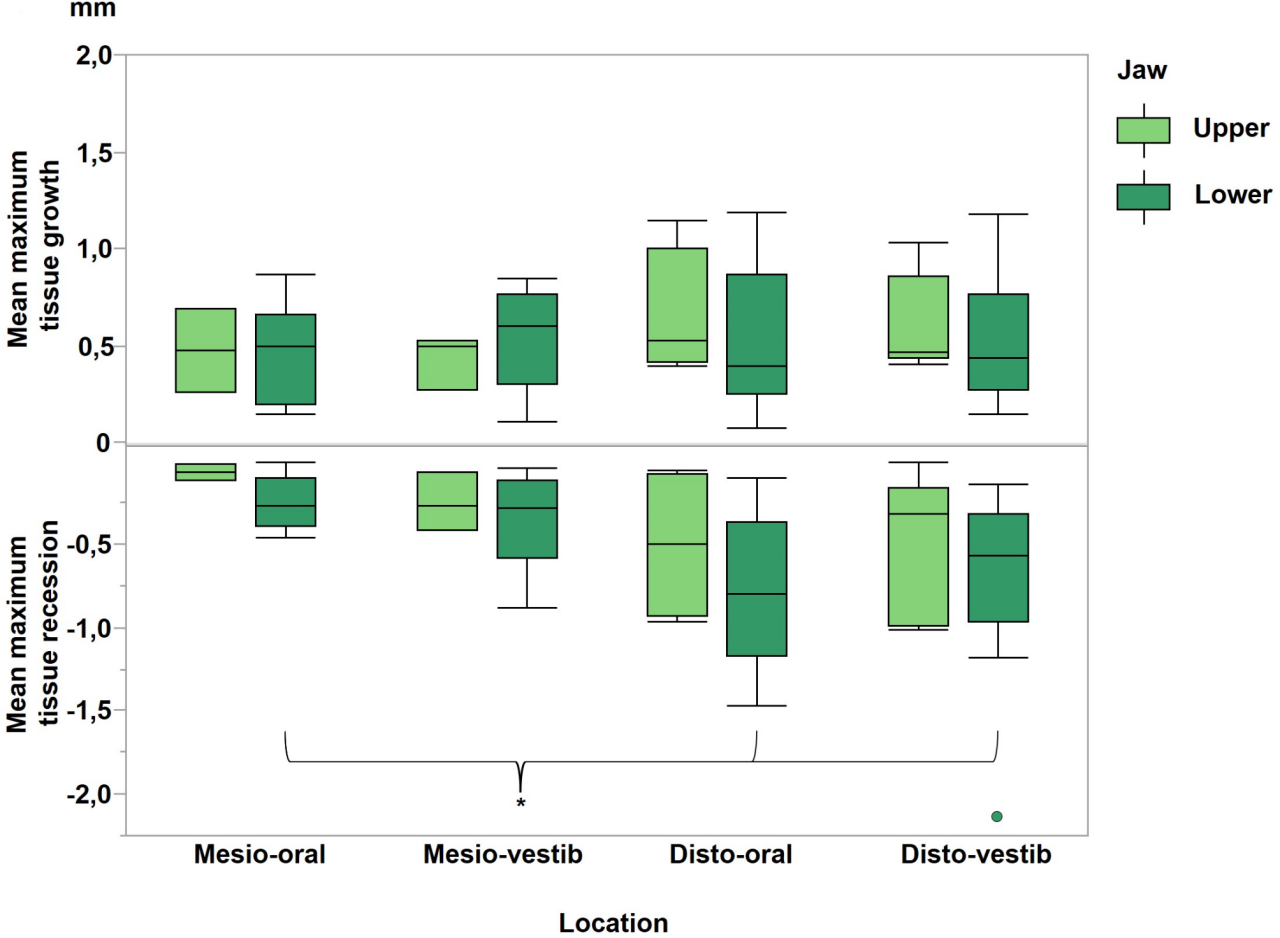
Mean maximum soft tissue changes for four various papillae regions

With regards to papillae profile, a statistically significant soft tissue recession was observed both in the disto-oral area and in the disto-vestibular area compared to the mesio-oral region (Fig. 7).

### Secondary outcomes: influence of anamnestic parameters

During the implant healing phase, a significantly higher recession was observed in the lower than in the upper jaw. In the delivery phase and up to the 12 months follow-up a significantly higher recession was observed in men than in women in the conical group and vice-versa in butt-joint group (Table 1). Any other anamnestic data did not influence on the soft tissue dimensions.

**Table 1:** Influence of various anamnestic parameters on the soft tissue changes during the implant healing phase (post-op – pre-exposure) in conical and plane implant connection types. Statistically significant difference was observed between the lower and upper jaw in the conical connection type group

	Maximal soft tissue gain				Maximal soft tissue recession			
	Implantat System				Implantat System			
	Butt-joint		Conical		Butt-joint		Conical	
**Sex**	**Mean**	**SD**	**Mean**	**SD**	**Mean**	**SD**	**Mean**	**SD**
male	0,92	0,90	0,58	0,55	-1,79	0,73	-1,19	0,57
female	0,88	0,69	0,32	0,16	-1,43	0,53	-1,00	0,38
**Region**								
upper jaw	0,37	0,21	0,37	0,19	-1,30	0,33	-0,97	0,51
lower Jaw	1,11	0,84	0,70	0,68	-1,77	0,71	-1,37	0,50
**Brushing / Day**								
</=2	0,87	0,73	0,37	0,18	-1,59	0,69	-1,05	0,46
> 2	1,10	1,43	0,87	0,79	-1,89	0,44	-1,36	0,66
**Rinsing**								
yes	0,90	0,82	0,57	0,56	-1,84	0,54	-1,25	0,57
no	0,90	0,82	0,36	0,11	-1,26	0,73	-0,83	0,19
**Flossing**								
yes	1,00	0,86	0,52	0,48	-1,75	0,70	-0,98	0,47
no	0,53	0,25	0,52	0,54	-1,22	0,13	-1,35	0,55
**Stress**								
medium	1,00	0,81	0,54	0,51	-1,67	0,69	-1,13	0,55
high	0,33	0,20	0,33	0,08	-1,43	0,39	-1,26	0,40
**Alcohol consump.**								
< 1x / week	0,80	0,60	0,53	0,47	-1,64	0,71	-1,21	0,58
≥ 1x /week	1,04	1,03	0,50	0,54	-1,63	0,63	-1,07	0,48
**Periodontitis therapy**								
yes	0,57	0,32	0,64	0,51	-1,02	0,17	-1,17	0,52
no	0,96	0,84	0,44	0,48	-1,74	0,65	-1,13	0,56
**Gingival type**								
thick flat	0,84	0,76	0,45	0,43	-1,60	0,72	-1,11	0,44
thin scalloped	1,13	1,01	0,67	0,63	-1,76	0,38	-1,23	0,73

## Discussion

The significantly higher recession for the butt-joint group and significantly higher growth of soft tissues for conical connection group was observed. For this reason, the null hypothesis was rejected.

There may be two potential reasons for this observation. The first one is attributed to the crestal bone level and potential remodeling of the bone. An aggressive remodeling might occur due to a very high position of the implant. This way the supracrestal dimension of 3–4 mm may be disrespected, so the bone tries to compensate and adjust it, causing bone loss [19, 20]. A bone loss itself influences the soft tissue profile. In the present study, care was taken to place each type of implant according to manufacturer recommendations and maintaining the 3–4 mm distance from the soft tissue margin. Thus, in case of conical connection, the implants were paced subcrestally. In case of butt-joint connection most of the implants were place paracrestally. If there was a need to place the butt-joint implant deeper, the profile bur was used to adjust the crestal bone portion. If this step would be neglected, this might have caused a greater remodeling in the follow-up phase.

The integrated platform switch in case of conical connection is generally beneficial regarding the interaction with the crestal bone [21]. This phenomenon could be attributed to the specific design of conical implant connections. Specifically, the integrated platform-switch, which allows for a tapered connection between the implant and crown, could have a significant impact on soft tissue growth [22]. This design allows to increase the distance between the implant-abutment connection and the surrounding bone, which is normally approximately about one millimeter. This aids a better crestal bone stability. In case of butt-joint connection implant systems, the connection line is directly in touch with the peri-implant bone and soft tissue. This could lead to greater bone resorption, which in turn could result in greater recession. Thus, a conical connection may promote stability and tissue response by facilitating a more harmonious interaction between the implant and the surrounding tissue [23]. This might be due the reduction of mechanical stress which is achieved by platform switching [11]. Micromovements between the implant and abutment are also reduced which in turn reduces the stress on the surrounding tissue and can therefore reduce the likelihood of inflammation [21,23–25].

During the implant healing phase, the soft tissues around implants in the lower jaw tend to recede more pronouncedly than in the upper jaw. This finding can have important implications for the planning and execution of dental implant procedures and may require specific considerations and measures to optimize aesthetic and functional outcomes for patients in both jaws. A possible explanation for the observed differences in soft tissue recession could lie in the biomechanical loads and forces that occur in the mandible compared to the maxilla. The structural load caused by masticatory movements and the resulting stress on the implants could be higher in the mandible. Furthermore, a less amount of keratinized gingiva may also explain a lower soft tissue stability in the lower jaw.

The analysis of marginal bone loss can provide valuable insights into the long-term stability and survival of implants. However, it must be acknowledged that direct measurements of marginal bone loss were not conducted in our study. This constitutes a limitation and could potentially influence the interpretation of our results. Therefore, future research endeavors should encompass a more detailed assessment of marginal bone loss to better comprehend the potential effects of differing implant geometries and to determine their clinical relevance more accurately.

The higher recession of the papillae in the disto-oral and disto-vestibular areas compared to the mesio-oral area, raises interesting questions regarding the underlying causes and potential clinical implications. The findings suggest that specific measures for preserving soft tissue in the mandible, particularly in the posterior region, may be necessary. This could involve augmentation procedures using connective tissue grafts or xenogeneic matrices. In general, these observations provide valuable insights into soft tissue dynamics in the mandible and could influence clinical decisions in planning and implementing implant treatments. Future research could aid a better understanding of the underlying mechanisms and develop optimal strategies for soft tissue care in this specific context. In literature there are several publications that have dealt with the crucial aesthetic role of the papillae around implants [26–29].

The anamnestic factors showed only limited influence on the statistics, which can be attributed to the following reasons: Before implant placement, patients were screened, resulting in the exclusion of patients with risk factors such as poor oral hygiene, heavy smoking, periodontitis, etc. The final scan was conducted 12 months postoperatively which means that on average 7 months had elapsed between the placement of the implant crown and the final scan. In order to detect any potential influence of the mentioned anamnestic factors on soft tissue a longer observational period might be needed. Another intriguing aspect is the potential role of the patients` oral hygiene and smoking behaviour in relation to the observed differences. Future studies should investigate the influence of smoking on the dimensional changes of peri-implant soft tissues.

One study limitation is the relatively small sample size of forty patients. The effectiveness and generalizability of the findings may be influenced by the limited number of participants. The follow-up period of 12 months, while providing insights into short-term outcomes, may not capture the long-term performance of the implants. Longer follow-up periods would be beneficial to assess the stability and success rates of both butt-joint and conical implant connections over an extended duration.

The volumetric calculations were not feasible, so only linear discrepancies could be determined. This limitation arises from intraoral scans, which, despite being three-dimensional, do not inherently provide volumetric representations. To ensure precise volume measurements it would be necessary to complement intraoral scans with CBCT scans for each patient. This additional step would enable the deduction of the pure soft tissue changes, allowing for a more accurate assessment of volumetric alterations over time. However, this approach would be ethically questionable especially with a view to radiation dose from CBCTs.

Further limitation of the study are the absence of a placebo-control group and the fact that only two different implant systems were compared to each other. Utilization of butt-joint implant connection with integrated platform switch would be also advantageous. Furthermore, the present study utilized a closed healing approach. A direct installation of the healing abutment straight after implant placement may have its own influence on dimensional changes of the peri-implant soft tissues after the healing phase. Moreover, some studies reported the use of individualized and patient-specific healing abutments, which may also positively influence soft tissue reliefs and specifically aid a better papillae preservation [30]. These aspects should be addressed in future research.

## Conclusion

Within the limitations of the present clinical study, the conical connection implant system exhibited more soft tissue gain and less recession, compared to the butt-joint connection type. The soft tissue recession was higher in the mandible compared to the maxilla. A higher loss of soft tissue was observed in the distal papilla than in the mesial one. In light of these findings, the surgeon should carefully weigh the choice of the implant system. This regional variability suggests that targeted measures in treatment planning are necessary to preserve the integrity of soft tissue, especially in anterior regions.

Acknowledgment.

## Data Availability

No datasets were generated or analysed during the current study.

## Abbreviations

DICOM: Digital Imaging and Communications in Medicine
STL: Standard Tesselation Languange
CBCT: Cone Beam Computer Tomography

## References

[1] Warreth A, McAleese E, McDonnell P, Slami R, Guray SM. Dental implants and single implant-supported restorations. J Ir Dent Assoc. 2013;59(1):32–43. 23539970

[2] Wang Y, Bäumer D, Ozga A-K, Körner G, Bäumer A. Patient satisfaction and oral health-related quality of life 10 years after implant placement. BMC Oral Health. 2021;21(1). 10.1186/s12903-020-01381-3 PMC7807859 33446161

[3] Hebel K, Gajjar R, Hofstede T. Single-tooth replacement: bridge vs. implant-supported restoration. J Can Dent Assoc. 2000;66(8):435–8. 11040527

[4] Gomez-Meda R, Esquivel J, Blatz MB. The esthetic biological contour concept for implant restoration emergence profile design. J Esthetic Restor Dentistry. 2021;33(1):173–84. 10.1111/jerd.12714 33470498

[5] Kadkhodazadeh M, Amid R, Kermani ME, Mirakhori M, Hosseinpour S. Timing of soft tissue management around dental implants: a suggested protocol. Gen Dent. 2017;65(3):50–6. 28475086

[6] Siegenthaler M, Strauss FJ, Gamper F, Hämmerle CHF, Jung RE, Thoma DS. Anterior implant restorations with a convex emergence profile increase the frequency of recession: 12-month results of a randomized controlled clinical trial. J Clin Periodontol. 2022;49(11):1145–57. 35817419 10.1111/jcpe.13696 PMC9804465

[7] Tavelli L, Barootchi S, Avila-Ortiz G, Urban IA, Giannobile WV, Wang HL. Peri‐implant soft tissue phenotype modification and its impact on peri‐implant health: A systematic review and network meta‐analysis. J Periodontol. 2021;92(1):21–44. 32710810 10.1002/JPER.19-0716

[8] Gomez-Meda R, Esquivel J, Blatz MB. The esthetic biological contour concept for implant restoration emergence profile design. J Esthet Restor Dent. 2021;33(1):173–84. 33470498 10.1111/jerd.12714

[9] Jepsen S, Berglundh T, Genco R, Aass AM, Demirel K, Derks J, et al. Primary prevention of peri-implantitis: Managing peri-implant mucositis. J Clin Periodontol. 2015;42:S152–7. 25626479 10.1111/jcpe.12369

[10] Laleman I, Lambert F. Implant connection and abutment selection as a predisposing and/or precipitating factor for peri-implant diseases: A review. Clin Implant Dent Relat Res. 2023;25(4):723–33. 36825512 10.1111/cid.13185

[11] Hsu YT, Lin GH, Wang HL. Effects of Platform-Switching on Peri-implant Soft and Hard Tissue Outcomes: A Systematic Review and Meta-analysis. Int J Oral Maxillofac Implants. 2017;32(1):e9–24. 28095526 10.11607/jomi.5140

[12] Cheng GL, Leblebicioglu B, Li J, Chien HH. Soft tissue healing around platform-switching and platform-matching single implants: A randomized clinical trial. J Periodontol. 2020;91(12):1609–20. 32474935 10.1002/JPER.20-0030

[13] Mancini L, Galarraga-Vinueza ME, Barootchi S, Tavelli L. 3D surface defect map for characterising the buccolingual profile of peri-implant tissues. Int J Oral Implantol (Berl). 2023;16(2):105–13. 37158180

[14] Ruales-Carrera E, Pauletto P, Apaza-Bedoya K, Volpato CAM, Özcan M, Benfatti CAM. Peri-implant tissue management after immediate implant placement using a customized healing abutment. J Esthet Restor Dent. 2019;31(6):533–41. 31268244 10.1111/jerd.12512

[15] Thoma DS, Naenni N, Figuero E, Hämmerle CHF, Schwarz F, Jung RE, et al. Effects of soft tissue augmentation procedures on peri-implant health or disease: A systematic review and meta-analysis. Clin Oral Implants Res. 2018;29(Suppl):32–49. 29498129 10.1111/clr.13114

[16] Thoma DS, Buranawat B, Hämmerle CH, Held U, Jung RE. Efficacy of soft tissue augmentation around dental implants and in partially edentulous areas: a systematic review. J Clin Periodontol. 2014;41(Suppl 15):S77–91. 24641003 10.1111/jcpe.12220

[17] Ramanauskaite A, Obreja K, Müller KM, Schliephake C, Wieland J, Begic A, et al. Three-dimensional changes of a porcine collagen matrix and free gingival grafts for soft tissue augmentation to increase the width of keratinized tissue around dental implants: a randomized controlled clinical study. Int J Implant Dent. 2023;9(1):13. 37326686 10.1186/s40729-023-00482-2 PMC10275822

[18] Müller P, Ender A, Joda T, Katsoulis J. Impact of digital intraoral scan strategies on the impression accuracy using the TRIOS Pod scanner. Quintessence Int. 2016;47(4):343–9. 26824085 10.3290/j.qi.a35524

[19] Mattheos N, Vergoullis I, Janda M, Miseli A. The Implant Supracrestal Complex and Its Significance for Long-Term Successful Clinical Outcomes. Int J Prosthodont. 2021;34(1):88–100. 33570524 10.11607/ijp.7201

[20] Puisys A, Janda M, Auzbikaviciute V, Gallucci GO, Mattheos N. Contour angle and peri-implant tissue height: Two interrelated features of the implant supracrestal complex. Clin Exp Dent Res. 2023;9(3):418–24. 36988518 10.1002/cre2.731 PMC10280599

[21] Gupta S, Sabharwal R, Nazeer J, Taneja L, Choudhury BK, Sahu S. Platform switching technique and crestal bone loss around the dental implants: A systematic review. Ann Afr Med. 2019;18(1):1–6. 30729925 10.4103/aam.aam_15_18 PMC6380118

[22] Farronato D, Manfredini M, Farronato M, Pasini PM, Orsina AA, Lops D. Behavior of Soft Tissue around Platform-Switched Implants and Non-Platform-Switched Implants: A Comparative Three-Year Clinical Study. J Clin Med. 2021;10(13). 10.3390/jcm10132955 PMC8269426 34209354

[23] Rodrigues VVM, Faé DS, Rosa C, Bento VAA, Lacerda M, Pellizzer EP, et al. Is the clinical performance of internal conical connection better than internal non-conical connection for implant-supported restorations? A systematic review with meta-analysis of randomized controlled trials. J Prosthodont. 2023;32(5):382–91. 36700461 10.1111/jopr.13655

[24] Schmitt CM, Nogueira-Filho G, Tenenbaum HC, Lai JY, Brito C, Döring H, et al. Performance of conical abutment (Morse Taper) connection implants: a systematic review. J Biomed Mater Res A. 2014;102(2):552–74. 23533139 10.1002/jbm.a.34709

[25] Ackermann KL, Barth T, Cacaci C, Kistler S, Schlee M, Stiller M. Clinical and patient-reported outcome of implant restorations with internal conical connection in daily dental practices: prospective observational multicenter trial with up to 7-year follow-up. Int J Implant Dent. 2020;6(1):14. 32266497 10.1186/s40729-020-00211-z PMC7138872

[26] Ramanauskaite A, Sader R. Esthetic complications in implant dentistry. Periodontol 2000. 2022;88(1):73–85. 35103323 10.1111/prd.12412

[27] Chow YC, Wang HL. Factors and techniques influencing peri-implant papillae. Implant Dent. 2010;19(3):208–19. 20523177 10.1097/ID.0b013e3181d43bd6

[28] Stefanini M, Marzadori M, Tavelli L, Bellone P, Zucchelli G. Peri-implant Papillae Reconstruction at an Esthetically Failing Implant. Int J Periodontics Restor Dent. 2020;40(2):213–22. 10.11607/prd.4296 32032405

[29] Smukler H, Castellucci F, Capri D. The role of the implant housing in obtaining aesthetics: generation of peri-implant gingivae and papillae–Part 1. Pract Proced Aesthet Dent. 2003;15(2):141–9. quiz 50. 12772631

[30] Teślak M, Ziemlewski A, Foltyn I, Ordyniec-Kwaśnica I, Drogoszewska B. Development of Custom Anatomic Healing Abutment Based on Cone-Beam Computer Tomography Measurement on Human Teeth Cross-Section. Mater (Basel). 2021;14(16). 10.3390/ma14164570 PMC8401006 34443093

